# Transcription level differences in *Taxus wallichiana* var. *mairei* elicited by Ce^3+^, Ce^4+^ and methyl jasmonate

**DOI:** 10.3389/fpls.2022.1040596

**Published:** 2022-11-10

**Authors:** Na Han, Wen-ji Geng, Jian Li, Shu-ting Liu, Jie Zhang, Yi-jie Wen, Huai-hua Xu, Meng-yuan Li, Yan-ru Li, Pei-pei Han

**Affiliations:** Key Laboratory of Industrial Fermentation Microbiology, Ministry of Education, State Key Laboratory of Food Nutrition and Safety, College of Biotechnology, Tianjin University of Science and Technology, Tianjin, China

**Keywords:** *T. mairei*, transcriptome, taxol biosynthesis, cerium, MJ

## Abstract

Taxol is a precious and effective anticancer drug. Cerium and methyl jasmonate (MJ) have been shown to increase the yield of taxol in taxus cells. However, the mechanisms of cerium-mediated and MJ-mediated taxol biosynthesis remain unknown. RNA-Seq was applied to study the overall regulation mechanism of cerium and MJ on taxol biosynthesis and analyze the differences among *T. mairei* cells elicited by Ce^3+^, Ce^4+^ and MJ on transcriptional level . Using sequence homology, 179 unigenes were identified as taxol synthesis genes. Under the condition of 100 μM MJ, taxol synthesis genes were up-regulated. Notably, taxol synthesis genes were down-regulated expression at 1 mM Ce^3+^ and 1 mM Ce^4+^. Differential expression genes involved in some related functions were analyzed, such as MAPK signaling pathway and plant-pathogen interaction. Sequence alignment and phylogenetic analysis of nine differentially expressed WRKYs in our data were carried out.

## Introduction

Taxus, a genus of gymnosperms, contains at least 14 species (Hao et al., 2008). *T. mairei* is a member of taxus, which is a tall evergreen tree mainly distributed in southeast China (Wang et al., 2019). *T. mairei* can produce many chemicals with pharmaceutical properties. Among them, Taxol^®^ was reported as a quite promising anticancer drug in 1964 (Fang and Liang, 2005). Taxol is a plant protectant , which is a diterpenoid compound (Wu and Yuan, 2000).

At present, taxol production still depends on natural bark extraction or semi-synthesis of taxus extraction precursors such as baccatin III (Mutanda et al., 2021). Paclitaxel drug has been in short supply for a long time and the price is high, so developing a biosynthesis method with low cost and high yield is particularly important (Wang et al., 2013; Kuang et al., 2016). The basic framework of taxol biosynthesis pathway had been established, and most of the enzymes involved in the synthesis had been cloned (Kuang et al., 2016). However, the biosynthesis pathway of taxol is unclear. Therefore, further exploration is needed to realize the continuous supply of taxol through biosynthesis. (Wen et al., 2016; Abdallah et al., 2019; Wang et al., 2021).

Elicitors can stimulate plant cells or serve as a messenger to induce the activation of the plant defense system. Elicitors not only leads the primary metabolism of the plant cells to flow in the direction of secondary metabolism but also have little influence on the culture system and finally promotes the synthesis of the target product (Mani et al., 2021).

A previous study has demonstrated that light metals such as cerium and lanthanum are very effective non-biological elicitors to promote the biosynthesis of taxol (Mithofer et al., 2004). Studies confirmed that 0.1 mM Ce^4+^ can effectively improve the soluble protein synthesis and cell viability of *Taxus cuspidata* cells in suspension culture and increase taxol biosynthesis and release, while 1 mM Ce^4+^ can significantly induce apoptosis of *Taxus cuspidata* cells (Yingjin et al., 1998; Ge et al., 2005). It was found that Ce^4+^ can effectively promote the hydrolysis of phosphoglyceride and the cleavage of nucleotide chain (Sun et al., 2003). The Ce^3+^ can promote the germination of pollen grains, the growth of pollen tubes and the generation of safranin in tomato callus (Chen et al., 2004). Using rare earth as a unique biological effect to improve the synthesis of taxol is of great significance for large-scale industrial production in the future. The binding of rare earth to certain molecules on the cell membrane is well known. However, how this complex further affects the transcription of defense groups needs to be further confirmed.

In addition, previous studies have reported that the addition of MJ to the suspension culture of taxus cells can significantly induce the production of taxol (Ketchum et al., 1999; Khosroushahi et al., 2006). Therefore, taxus cells with different elicitors and elicitation time can be used as a differential expression system for studying taxol biosynthesis. Moreover, compared with the transcriptome of *T. mairei* responding to MJ, the effect of cerium on the transcriptome of *T. mairei* can be better clarified.

With the advantages of high throughput, high sensitivity, low cost and wide application, RNA-seq has become the main method in the research field of transcriptome (Sharma et al., 2018; Pop-Bica et al., 2022). RNA-seq has been applied to understand various aspects of taxol synthesis. In 2010, Lee et al. (Lee et al., 2010) reported the first transcriptome of taxus with cambium meristem cells from *Taxus cuspidata*. Li et al. (Li et al., 2012) studied the early response of *Taxus chinensis* cells elicited by methyl jasmonate (MJ) and found that a series of TFs activated by exogenous MJ, such as MYB, bHLH, ERF, AP2 and MYC, may be involved in the regulation of gene expression in taxol synthesis pathway. Yu et al. (Yu et al., 2017)studied the difference in transcription level between *Taxus media* and *T. mairei*, and proposed that the difference of taxane compounds content may be attributed to the differential expression of candidate genes involved in taxane biosynthesis pathway. However, the genome of taxus is complex, and there is still a lack of comprehensive understanding of the regulation of gene expression profile of taxol biosynthesis. At present, whole genome sequencing and related transcriptomics research are also rare, all of which indicate that the research of new generation of high-throughput sequencing technology in taxus has not yet arisen (Cusido et al., 2014).

In the present study, *T. mairei* cells elicited by Ce^3+^, Ce^4+^ and MJ were compared based on data generated by the same RNA-Seq method, and the regulation mechanism of cerium and MJ in taxol biosynthesis were investigate and the differences among *T. mairei* cells elicited by Ce^3+^, Ce^4+^ and MJ on transcriptional level were analyzed. And we expected that the biological information about the new DEGs, WRKYs and taxol synthesis-related pathways in this study would provide a basis for further research on cerium and MJ response network and regulation of taxane biosynthesis in *Taxus chinensis*.

## Materials and methods

### Plant materials and treatment condition

The cells used in the experiment were callus of *T. mairei*. The tender stems of T. mairei were disinfected with 75% ethanol and 5% sodium hypochlorite successively. The disinfected tender stems of *T. mairei* were cut into small sections and obliquely inserted into modified B5 solid medium. Callus began to grow after half a month. The cells were inoculated on modified B5 solid medium and subcultured once every 30 days at 25°C in the dark. The cells were activated in B5 liquid medium and cultured in liquid suspension at 25°C and 110 rpm in the dark. The subculture was conducted once every 10-12 d and the flask culture was conducted step by step. The specific steps were as follows: in the first stage of culture, about 3 g subcultured cells were inoculated into a 250 ml triangular flask containing 50 ml of fresh culture medium; During the second and third-stage culture, a part of the culture solution in the previous-stage culture was poured out, and fresh culture medium was supplemented to 50 ml; The fourth and fifth stages of culture were conducted in a 500 ml triangular flask with the medium changed, and the culture volume was maintained at about 150 ml. The fifth generation of liquid cells with uniform growth was sub-packaged into a 250 ml triangular flask containing 50 mL of fresh medium, and the inoculated cells weighed about 2.5-3.5 g cells (fresh weight). Subsequently, 1 mM Ce(NO_3_)_3_, 1 mM Ce(NH_4_)_2_(NO_3_)_6_, 100 μM MJ and an equal volume of solvent as control were added to the cultures respectively. The experiment was performed using three biological replicates for each sample.Taxanes extraction and HPLC analysis

The dried *T. mairei* cells (about 200 mg) were powdered and ultrasonicated for 10 min twice in mixed cyclohexane and methanol (1:1, v/v), then extracted three times with methylene chloride. Following centrifugation, the supernatant from the samples was filtered through a 0.2 μm polymeric filter. Filtered medium samples were used to extract taxol in a separatory funnel along with an equivalent volume of methylene chloride. Before HPLC analysis, the aqueous phase was eliminated, the methylene chloride phase was collected and allowed to evaporate at room temperature, and the remaining material was then resuspended in 1 ml of methanol and filtered through a 0.2 μm polymeric filter. The column, which was 250 mm long and 4.6 mm in diameter, was packed with Kromasil C_18_ 5 μm and washed at a rate of 1 mL per minute with a 65:35 (v/v) combination of methanol and water. At 227 nm, the detection was made. (Yuan et al., 2001)The content of taxane was calculated by an external standard method, and the pure products of taxol, 10-deacetylbaccatin III (10-DAB) and baccatin III were taken as the reference substances.

### Total RNA isolation and cDNA library construction

According to the previous report (Nims et al., 2006; Yang et al., 2006; Patil et al., 2012; Mao et al., 2018) using qRT-PCR on the time of taxol synthesis gene and some important enzyme response elicitors, we optimized the sampling time to 6h and 24h to study the changes in the transcription level of *T. mairei* cells. For the RNA-Seq, samples were taken at 6 h and 24 h after 1 mM Ce(NO_3_)_3_, 1 mM Ce(NH_4_)_2_(NO_3_)_6_ and 100 μM MJ addition. After the total RNA extraction (three biological replicates for each sample) and DNase I treatment, magnetic beads with Oligo (dT) were used to isolate mRNA(for eukaryotes) or by removing rRNAs from the total RNA(for prokaryotes). Mixed with the fragmentation buffer, the mRNA was fragmented into short fragments. Then cDNA was synthesized using the mRNA fragments as templates. Short fragments were purified and resolved with EB buffer for end reparation and single nucleotide A (adenine) addition. After that, the short fragments were connected with adapters. After agarose gel electrophoresis, the suitable fragments were selected for the PCR amplification as templates. During the QC steps, Agilent 2100 Bioanalyzer and ABI StepOnePlus Real-Time PCR System were used in quantification and qualification of the sample library.

### Illumina sequencing and *de novo* assembly

The Illumina Hiseq4000 sequencing platform was used to carry out the RNA sequencing. Transcript data of each sample was assembled from scratch using Trinity (Grabherr et al., 2011), and TGICL (Pertea et al., 2003) was used for unigenes clustering and redundancy reduction to produce unigenes and transcripts. The sequences data reported in this study were archived in the Sequence Read Archive (SRA) with the accession number SRR20217393.

### Gene function annotation and differential expression analysis

Blastx was used to align unigenes sequences to the protein database NR, Swiss-Prot, KEGG and COG (Tatusov et al., 2003) (Evalue<1e-5), and blastn was used to align unigenes to the nucleic acid database nt (Evalue<1e-5). The protein with the highest sequence similarity was used as the function annotation information of the unigenes.

Using the results of NR annotation, GO function annotation for unigenes was obtained using Blast2GO (Conesa et al., 2005). Clean data obtained after filtration was respectively compared with the assembled unigenes through SOAPaligner v2.21 (Li et al., 2009), and the expression level of unigenes was calculated using RPKM (Mortazavi et al., 2008) (Reads per kB per Million fragments). The calculation formula is:


$$RPKM=\frac{10^{6}C}{NL/10^{3}}$$

In this formula, RPKM (A) is the expression of Unigene A, and C is the number of reads that are uniquely aligned to Unigene A, N is the total number of reads that are uniquely aligned to all Unigenes, and L is the number of bases on Unigene A.

The level of significant gene difference between the two samples was calculated using the detection method (Audic and Claverie, 1997) described by Audic S. et al. And the level of false positive rate was controlled using FDR (False Discovery Rate) [36]. The significantly differentially expressed genes were defined as the gene with FDR ≤ 0.001 and FC > 2.

To identify the most important biochemical metabolism, signal transduction Pathways and GO functional differences in which DEGs were involved, KEGG and GO functional enrichment analysis on DEGs was performed. Using a hypergeometric test, the functional categories of pathway and GO that were significantly enriched in differentially expressed genes were found. The level of the false-positive rate was controlled by FDR(False Discovery Rate) (Lange et al., 2003).

To eliminate the possible impact of the analysis of the significant difference in gene expression on the functional enrichment analysis of KEGG and GO, and identify the changing trends of all DEGs in the functional classifications of Pathway and GO, functional enrichment analysis on all DEGs in the KEGG and GO classifications were performed. The up-regulated genes were defined as fold change greater than 1, and the down-regulated genes were defined as fold change less than 1. The Chi-squared test was used to analyze the significance level of up-regulation or down-regulation of DEGs in the KEGG pathway and GO terms, and the level of false-positive rate was controlled by FDR(False Discovery Rate) (Lange et al., 2003).

### Identification of unigenes associated with the taxol biosynthesis pathway

All unigenes were matched to known genes involved in taxol production using the Blastx program. The gene in taxol synthesis was discovered as the best alignment of unigenes with Identity > 40%, Evalue > 10 e-7, and Coverage > 80%.

A gene associated with taxol production often has many unigenes. The differential expression of this gene was converted using the formula below:


$$Diffexp=log\left(\frac{\sum_{i=1}^{n}A\;RPKM}{\sum_{i=1}^{m}B\;RPKM}\right)/\text{log}\left(2\right)$$

In this formula, Diffexp represents the differential expression of a gene related to taxol synthesis; A RPKM represents the RPKM value of the corresponding unigene in Sample case A; B RPKM represents the RPKM value of the corresponding unigene in Sample control B; n: The number of unigenes from the same gene related to taxol synthesis in Sample case A; m: The number of unigenes in the same taxol synthesis-related gene in Sample control B.

### Sequence alignment and phylogenetic analyses

Using the maximum likelihood (ML) method in MEGA5 software, the WRKY phylogenetic tree was constructed and the WRKY domain sequences were aligned.

## Results and discussion

### Variation in the taxoid contents in elicited and unelicited *T. mairei* cells

**Figure 1:**
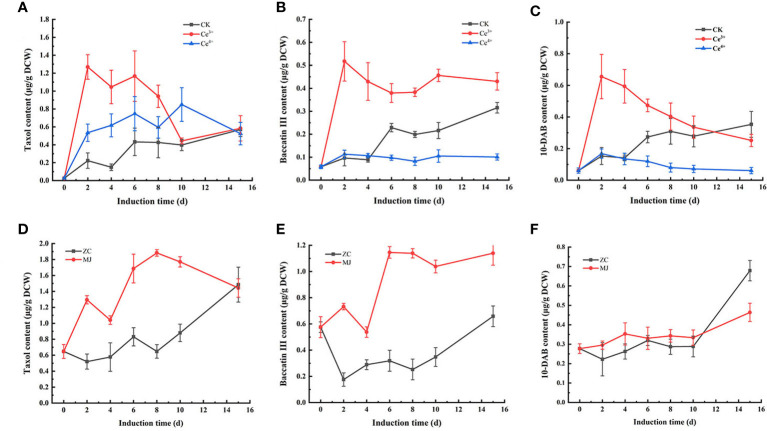
Changes of taxanes content including taxol **(A, D)**, baccatin III **(B, E)**, 10-DAB **(C, F)** in *T. mairei* cells elicited by Ce^3+^, Ce^4+^ and MJ. The sample ID of Ce^3+^, Ce^4+^ and MJ respectively represented the samples elicited by Ce^3+^, Ce^4+^ and MJ, CK and ZC represented unelicited samples (p<0.05).

**Figure 2:**
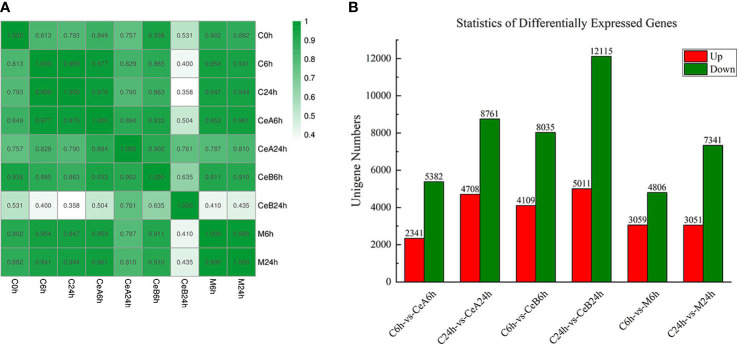
**(A)** Pearson correlation coefficients of all 9 samples. The expression level of each gene (the entire gene set) for each pair of samples was used to calculate the Pearson correlation coefficients, and the correlation coefficients between the two samples were visually displayed as a heat map. **(B)** The numbers of the up-regulated and down-regulated unigenes in the six comparisons.

The changes of taxol, baccatin III and 10-DAB contents in *T. mairei* cells elicited by 1 mM Ce^3+^, 1 mM Ce^4+^, and 100 μM MJ, respectively, were shown in **Figures 1**, **2**. The results showed that Ce^3+^, Ce^4+^ and MJ all obviously increased the synthesis of taxol. The three systems were used as differential expression systems for studying taxol biosynthesis. Therefore, cell samples elicited by 1 mM Ce^3+^, 1 mM Ce^4+^, and 100 μM MJ for 6 h and 24 h were selected for transcriptome sequencing.

### Transcriptome sequencing of elicited and unelicited *T. mairei* cells

The tender stem cell line of *T. mairei* was cultured in 1 mM Ce(NO_3_)_3_, 1 mM Ce(NH_4_)_2_(NO_3_)_6_ and 100 μM MJ elicitation system. Nine samples of elicited and unelicited cells cultured for 6 h and 24 h were selected for RNA transcription to form a library. The Illumina Hiseq4000 sequencing platform was used to generate a total of 62Gb of sequencing data, with approximate 60G of available data.

The Raw data for each sample was acquired. After base calling, preliminary quality analysis of the sequencing findings, and read filtering according to the processing guidelines, clean data can be acquired. In **Table 1**, quality data for each sample are presented. After filtration, each group received more than 40,000,000 reads.

**Table 1:** Statistics of Transcriptome Sequencing Data of *T. mairei*.

Sample ID	Total Raw Reads	Total Clean Reads	Read Length	Total Clean Nucleotides (nt)	Q20 (%)	GC (%)
C0	55329508	53877612	100	5387761200	98.40	45.26
C6h	61569240	59946702	100	5994670200	98.45	45.85
C24h	46073434	44859848	100	4485984800	98.35	46.01
CeA6h	91452112	89071954	100	8907195400	98.40	45.42
CeA24h	75508994	73591328	100	7359132800	98.60	45.26
CeB6h	81491064	79391952	100	7939195200	98.45	45.81
CeB24h	58596356	57123852	100	5712385200	98.55	45.2
M6h	73107524	71158468	100	7115846800	98.25	45.55
M24h	76392090	74318888	100	7431888800	98.35	46.12

The sample ID of CeAx, CeBx and Mx respectively represented the samples elicited at different times under the conditions of Ce^3+^, Ce^4+^ and MJ, Cx represented unelicited samples for different times.

The transcriptome sample data were assembled by trinity to create the contig sequence for each sample. The assembly results were then processed using sequence clustering software CDHIT for sequence splicing and redundancy removal to create a lengthy non-redundant unigene sequence. Sequence length and quantity were significant assessment factors for assembly quality. 88,326 unigenes were obtained through assembly, with an average length of 1076nt, N50 of 1811nt and GC ratio of 40.7%. The length of the unigenes was mainly distributed at 300 nt, followed by 400–1500 nt (**Supplementary Material 1**).

The functions of 51,516 unigenes were successfully annotated by NR, Swiss-Prot, KEGG, COG and GO. The number of unigenes annotated in each database was shown in **Supplementary Material 2**.

Based on the Nr annotation results, blast2GO software was used to analyze the GO function of the gene ontology. The GO information of each gene and the GO function classification were obtained, and distribution characteristics of gene function of species at the macro level were known. Among 51400 unigenes with Nr annotations, 35031 unigenes were annotated in GO. Under the three major categories of “molecular function”, “cell component” and “participation in biological processes”, all unigenes were divided into 49 functional groups by WEGO, of which 7 categories including cell, cell part, organelle, binding, catalytic, cellular process, metabolic process were the main categories (**Supplementary Material 3**).

### Differentially expressed gene analysis

The expression profiles of 88,326 unigenes were ultimately used for further analysis. **Figure 2A** showed the gene expression correlation between nine samples. The following differences were analyzed among six groups of samples: C6h-vs-CeA6h, C24h-vs-CeA24h, C6h-vs-CeB6h, C24h-vs-CeB24h, C6h-vs-M6h, C24h-vs-M24h. The statistical results of the differential expression of unigenes in *T. mairei* under different elicitation conditions were shown in **Figure 2B**.

**Figure 3:**
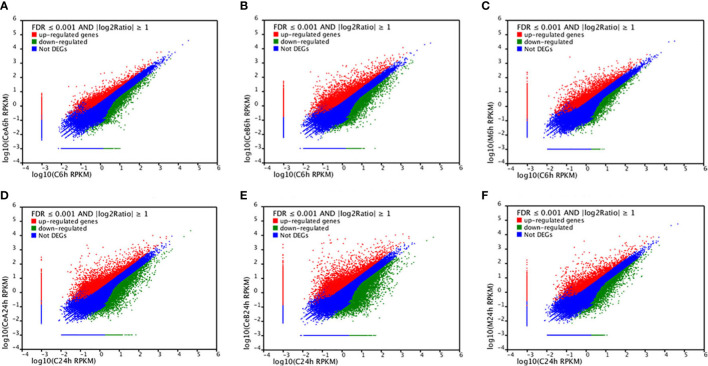
Expression levels in the six comparisons including C6h-vs-CeA6h **(A)**, C6h-vs-CeB6h **(B)**, C6h-vs-M6h **(C)**, C24h-vs-CeA24h **(D)**, C24h-vs-CeB24h **(E)** and C24h-vs-M24h **(F)**. The red points represent upregulated unigenes, the green points represent down-regulated unigenes, and the blue points represent non-DEGs (p<0.05).

**Figure 3** showed the changes in transcription level after elicitation of Ce^3+^, Ce^4+^ and MJ, showing that the number of up-regulated genes and down-regulated genes increased with the treatment time prolonged.

**Figure 4:**
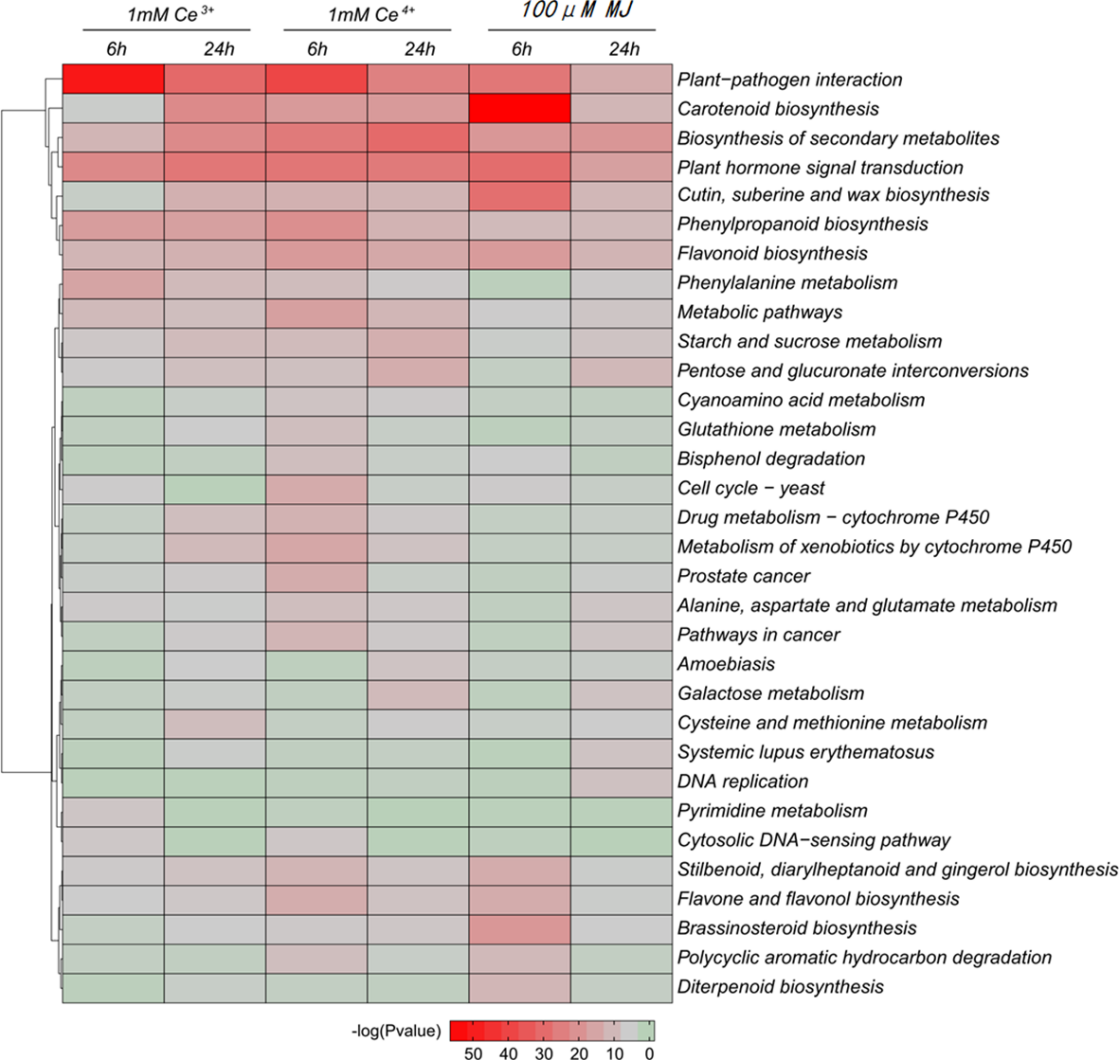
KEGG enrichment analysis of the DEGs in the six comparisons. The color indicates the significance level.

Differential genes were significantly enriched for plant-pathogen interaction and carotenoid biosynthesis based on KEGG enrichment analysis of DEGs (**Figure 4**). The results showed that the metabolic pathways of plant-pathogen interaction and carotenoid biosynthesis were significantly changed under the elicitation conditions of 1 mM Ce^3+^, 1 mM Ce^4+^, and 100 μM MJ in *T. mairei* cells.

The GO functional enrichment analyses of the DEGs in cellular component, molecular function and biological process were performed, respectively. The results showed that under the elicitation conditions of Ce^3+^, Ce^4+^ and MJ, cell wall, external encapsulating structure, oxidoreductase activity, catalase activity, diterpenoid and paclitaxel biosynthesis and other cellular functions changed significantly in *T. mairei*.

**Figure 5:**
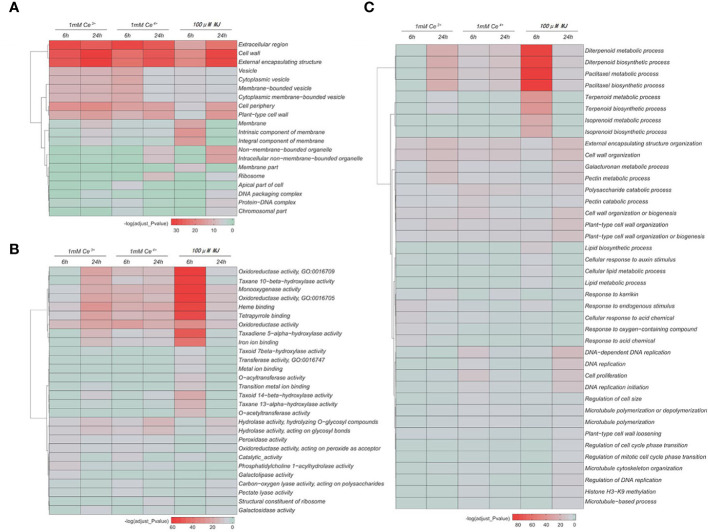
GO enrichment analysis of the DEGs in the six comparisons. **(A)** Cell composition **(B)** Molecular function **(C)** Biological process. The color indicates the significance level.

**Figure 6:**
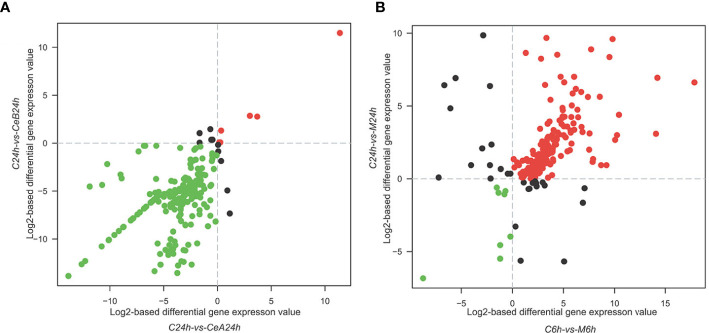
Comparison of gene expression during paclitaxel biosynthesis (GO:0042617) under different elicitation conditions. **(A)** The difference in gene expression of paclitaxel biosynthesis with 1 mM Ce^3+^ and 1 mM Ce^4+^ elicitation for 24 hours respectively. **(B)** The difference in gene expression of paclitaxel biosynthesis with 100 μM MJ elicitation for 6 and 24 hours.

The GO functional enrichment results of the differential genes showed that under the MJ elicitation, the paclitaxel biosynthesis process was significantly up-regulated and the redox process in the elicited *T. mairei* cells was significantly enhanced to provide energy supply for paclitaxel biosynthesis (**Figure 5**, **Figure 6B**). However, the biosynthesis of paclitaxel was significantly down-regulated at 1 mM Ce^3+^ and 1 mM Ce^4+^ elicitation (**Figure 5**, **Figure 6A**).

### DEGs involved in taxol biosynthesis

**Figure 7:**
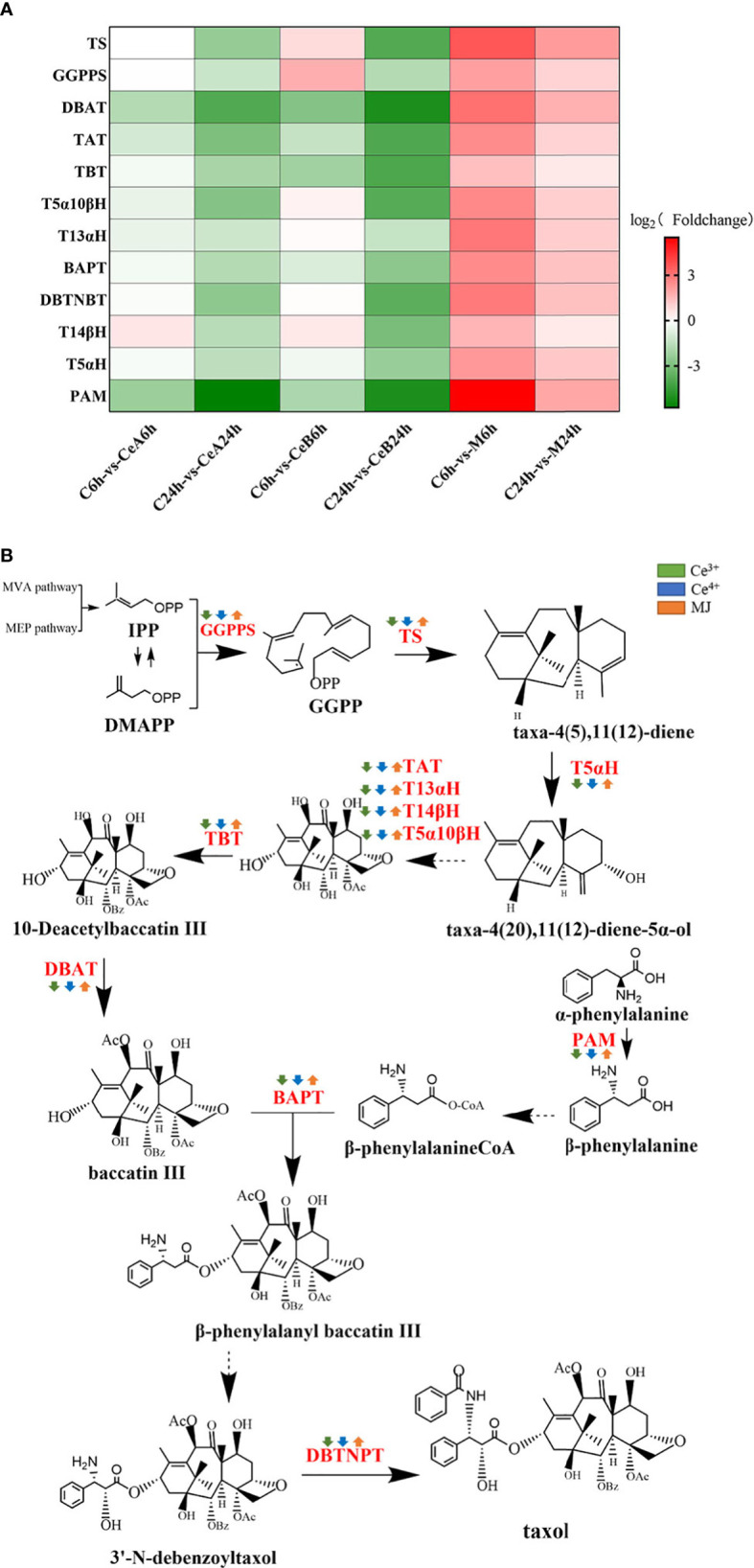
**(A)** Comparison of DEGs in taxol biosynthesis pathway. The color density indicated log_2_ (Foldchange). Red indicated the upregulation of gene expression and green indicated the downregulation of gene expression. **(B)** DEGs assigned to the taxol biosynthesis pathway. Green is elicited by Ce3+, blue is Ce4+, orange is MJ. The up arrows indicate the upregulation of gene expression; the down arrows indicate the downregulation of gene expression.

A total of 179 unigenes were identified as 12 taxol synthase coding genes by homologous comparison analysis. To elucidate the changes of DEGs in the taxol biosynthesis pathway after elicited by Ce^3+^, Ce^4+^ and MJ, differential enrichment analyses on gene expression of each gene were performed, and the results were shown in **Figure 7A**. The transcriptional expression changes of genes in taxol biosynthesis pathway were shown in **Figure 7B**. Gene expression levels of elicited cells showed obvious regularity compared with unelicited cells. Interestingly, the taxol synthesis genes were up-regulated under the MJ elicitation condition, but the taxol biosynthesis genes were down-regulated under the 1 mM Ce^3+^ and 1 mM Ce^4+^ elicitation conditions. It might be that Ce^3+^ and Ce^4+^ could significantly induce the *T. mairei* cells defensive response leading to apoptosis, and the expression of taxol synthesis gene in *Taxus chinensis* cells responded very quickly to cerium. A previous study (Yang et al., 2006) found that in the cell apoptosis elicited by Ce^4+^, an ERK-like kinase was rapidly activated at 5 min of elicitation, and continuously activated within 120min. After 240 min of elicitation, ERK-like was down-regulated and maintained until 48 h. Additionally, O_2_^-^ was rapidly elicited by Ce^4+^. The first burst of O_2_^-^ appeared in 3.7–4 h, and the second burst of O_2_^-^ appeared in 7 h. However, the specific mechanism of this interesting phenomenon need more study.

Taxol is a phytoalexin produced by the defensive reaction of taxus cells when infected by pathogenic bacteria, and the synthesis of taxol will be affected by the external environment and the cell state (Yuan et al., 2002). KEGG Pathway enrichment of the differential genes indicated that the plant-pathogen interaction pathway in *T. mairei* was significantly up-regulated after elicitation by Ce^3+^ and Ce^4+^. Hypersensitive response (HR) induced by the non-affinity interaction of plant pathogens is a typical programmed cell death model (Yang et al., 2006).

### Responsive genes involved in plant-pathogen interaction

**Figure 8:**
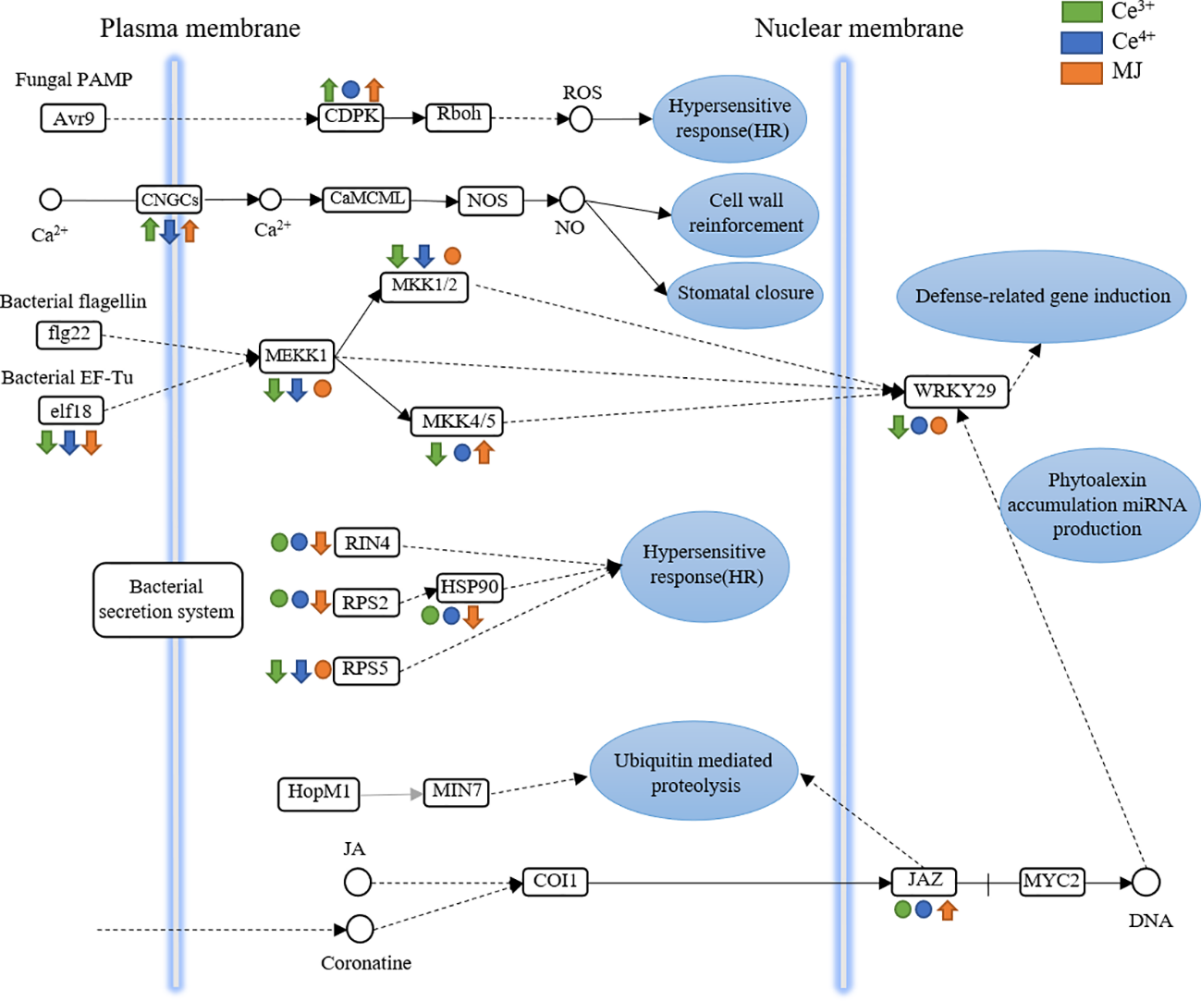
DEGs assigned to plant-pathogen interaction in C6h-vs-CeA6h, C6h-vs-CeB6h and C6h-vs-M6h. Green is elicited by Ce3+, blue is Ce4+, orange is MJ. The up arrows indicate the upregulation of gene expression; the down arrows indicate the downregulation of gene expression; the circles indicate unchanged expression.

The KEGG Pathway enrichment of DEGs indicated that plant-pathogen interaction pathway in *T. mairei* was significantly up-regulated after elicited by Ce^3+^, Ce^4+^ and MJ. Plant-pathogen interaction pathway was therefore analyzed. **Figure 8** shows the differential expression of proteins in the plant pathogen interaction pathway in C6h-vs-CeA6h, C6h-vs-CeB6h and C6h-vs-M6h.

The genes encoding CDPK were significantly up-regulated in C6h-vs-CeA6h and C6h-vs-CeB6h. The receipt and decoding of calcium signals were significantly regulated by the calcium-dependent protein kinase (CDPK), a class of key calcium signal receptors. Plant physiological activities such as growth and development, stress responses, and hormone signaling all depend on CDPKs. (Xiao et al., 2017; Li et al., 2022)

The genes encoding CNGCs were significantly up-regulated in C6h-vs-CeA6h and C6h-vs-M6h and down-regulated in C6h-vs-CeB6h. A class of non-selective cation channels called cyclic nucleotide-gated channels (CNGCs) was triggered by the direct binding of cyclic nucleotides like cAMP and cGMP to regulate cellular communication. These channels were mostly permeable to Ca^2+^. Plant growth, pathogen defense, and thermotolerance were all impacted by the CNGC family. (Kakar et al., 2017; Zhao et al., 2022)

The genes encoding elf8, MKK and MEKK were significantly down-regulated in C6h-vs-CeA6h and C6h-vs-CeB6h. These signals were transmitted into the nucleus and to transcription factors (WRKY29). The down-regulation of WRKY29 indicated the genes related to immune defense was down expression. The evolutionarily conserved MAPK signaling cascade, which was crucial for plant growth and development as well as various stress responses, was mainly composed of mitogen-activated protein kinase kinases (MKK). (Li et al., 2020)

The genes encoding JAZ were significantly up-regulated in C6h-vs-M6h. The primary repressors in the jasmonate signaling system were proteins from the Jasmonate ZIM-domain (JAZ) family, which were essential for plant growth, defenses, and stress responses. (Thines et al., 2007; Gfeller et al., 2010) Therefore, MJ might promote the expression of the JAZ-encoding gene and inhibit the JA signal transduction pathway. There were multiple regulators involved in the sensitive and dynamic process of JA recognition, signal transduction, and transcript reprogramming of downstream genes (Chini et al., 2007). Previous studies revealed the key role of JAZ protein in the biosynthesis of specific plant metabolites such as alkaloids, artemisinin and tanshinone. In *Artemisia annua*, AaJAZ8 negatively regulated artemisinin biosynthesis, the first line of defense against malaria, in response to JA elicitation (Ma et al., 2018). In *Salvia miltiorrhiza*, the overexpression of SmJAZ3 and SmJAZ9 reduced the content of tanshinone (Shi et al., 2016). Therefore, the JAZ gene had a wide range of roles in regulating adaptation to environmental challenges and in regulating the development and specific metabolism of different plants.

### Responsive genes involved in MAPK signaling pathway

Studies confirmed that 0.1 mM Ce^4+^ can effectively improve the soluble protein synthesis and cell viability of *Taxus cuspidata* cells in suspension culture and increase taxol biosynthesis and release, while 1 mM Ce^4+^ can significantly induce apoptosis of *Taxus cuspidata* cells (Yingjin et al., 1998; Ge et al., 2005). Taxol is a kind of plant protectant . Rare earth can stimulate taxus cells to improve taxol synthesis, indicating that rare earth can enhance the defensive response of taxus. (Yuan et al., 2002) Studies showed that Ce^4+^ induced *Taxus cuspidata* cells to activate O_2_^-^ generate through the NADPH oxidase pathway, which mediated the MAPK pathway or NO signaling molecule, and activated the downstream caspase-like enzyme through the signaling cascade (caspase-like family or mitochondrial pathway). The activation of three nucleases was a downstream event of signal transduction process, regulated by caspase3-like or other signaling molecules, and finally led to apoptosis. (Yuan, 2006)

There are varieties of MAPK pathways in plant cells. Each pathway is related and independent, and they play an important role in cell signal transduction (Sharma et al., 2021). In plants, the MAPK cascade is intertwined with other signal transduction pathways to form a molecular interaction network (Andreasson and Ellis, 2010).

**Figure 9:**
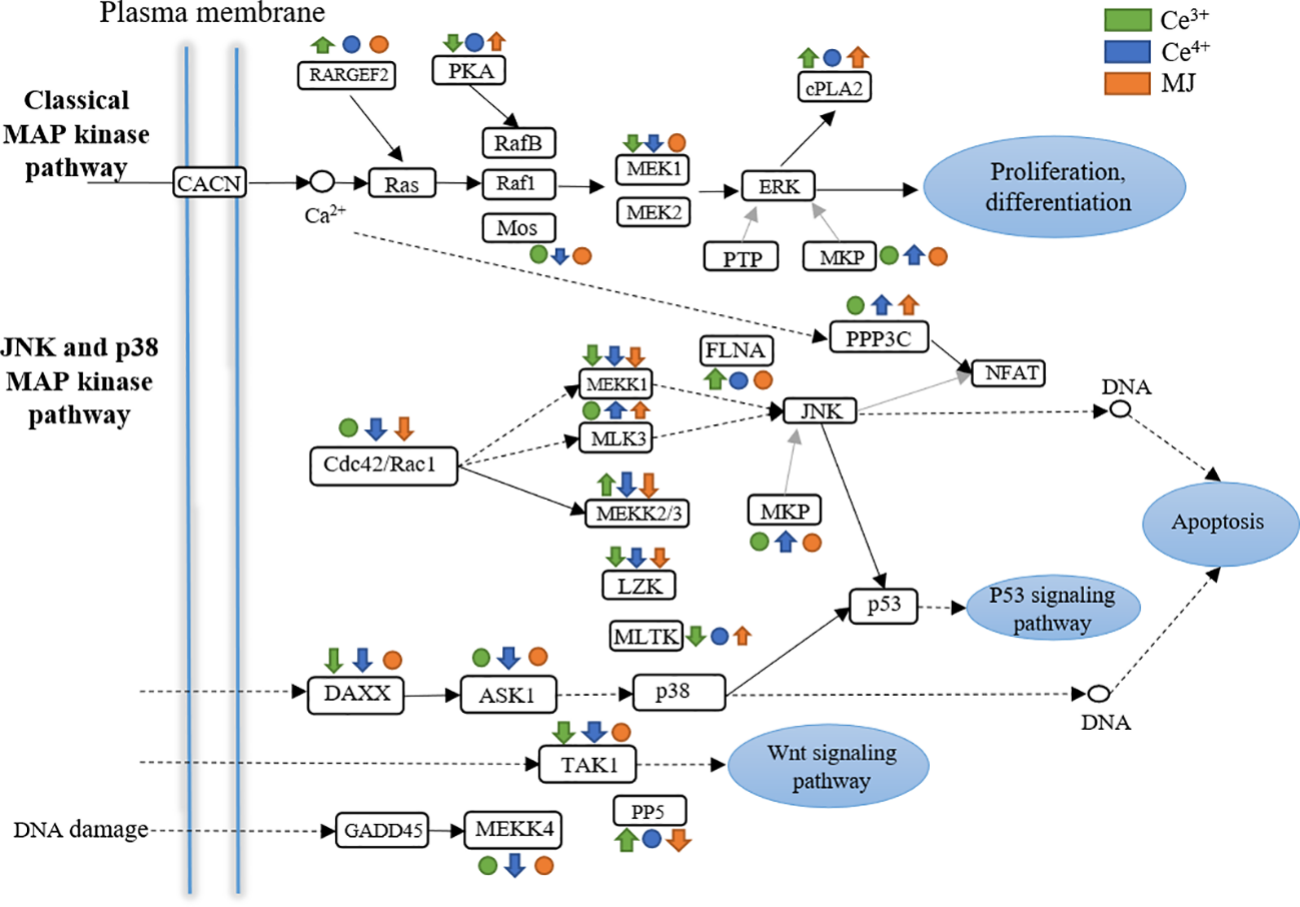
DEGs assigned to MAPK signaling pathway in C6h-vs-CeA6h, C6h-vs-CeB6h and C6h-vs-M6h. Green is elicited by Ce3+, blue is Ce4+, orange is MJ. The up arrows indicate the upregulation of gene expression; the down arrows indicate the downregulation of gene expression; the circles indicate unchanged expression.

**Figure 9** showed the differential expression of proteins in the MAPK pathway in C6h-vs-CeA6h, C6h-vs-CeB6h and C6h-vs-M6h. Genes encoding MKP, PPP3C and MLK3 were significantly up-regulated and genes encoding MEKK1, MEKK2/3, LZK, DAXX, ASK1, TAK1, etc. were significantly down-regulated in C6h-vs-CeB6h. Besides, *T. mairei* cells elicited by Ce^3+^ and MJ showed the same gene expression trend like that elicited by Ce^4+^.

MEKK1 protein is a key kinase activator for cell stress response. Activation of MEKK1 can stimulate a variety of reactions, including mitogen-activated protein (MAP) kinase and cell migration (Filipcik et al., 2020). MEKK2 and MEKK3 belong to the MEKK/STE11 subfamily that is widely expressed and effective activators of the NF-κB and MAPK pathways (Zhang et al., 2006). The Death-domain associated protein 6 (DAXX) is an evolutionarily conserved and widely expressed multifunctional protein, which is involved in many cellular processes, including transcription, cell proliferation, cell cycle regulation, Fas-induced apoptosis and many other events. In the nucleus, DAXX interacts with transcription factors, epigenetic modifiers, and chromatin-remodeling proteins (Bogolyubova and Bogolyubov, 2021). TAK1 functions as a mediator in the signaling pathway of transforming growth factor-beta (TGF-beta) superfamily members (Yamaguchi et al., 1995).

Diverse cellular functions in plants, including growth, development, biological and abiotic stress responses, are regulated by this network. For instance, MAPK in plants can target and regulate transcriptional factors including bZIP, MYB, MYC and WRKY under stress conditions (Zhang and Zhang, 2022).

### Regulation of the expression of WRKY

By stimulating the co-expression of many genes in the secondary metabolic pathway, TFs contribute significantly to the control of secondary metabolite production and accumulation (Hong et al., 2013). It has been reported that several transcription factor families, including WRKY, bHLH, and AP2/ERF, are involved in the production and accumulation of taxol (Dai et al., 2009; Li et al., 2013; Lenka et al., 2015; Zhang et al., 2015).

The conservative WRKYGQK motif in the WRKY domain is whence WRKY got its name (Wu et al., 2005). A group of plant-specific transcription factors (TFs) known as WRKY is crucial for pathogen defense, non-biological signaling, plant hormone signaling, and the control of secondary metabolism (Zhang et al., 2018). For example, AaWRKY1 regulated the amorpha-4,11-diene synthase, which was involved in the biosynthesis of artemisinin in Artemisia annua (Dongming Ma et al., 2009). GaWRKY1 regulated the sesquiterpene synthase CAD1 ((+)- δ-cadinene synthase -A) gene, which was involved in the regulation of the gossypol pathway in plant cotton (Xu et al., 2004). A study has found that TcWRKY1 can specifically interact with the promoter of the DBAT gene in paclitaxel biosynthesis. The other two WRKY TF, TcWRKY8 and TcWRKY47, significantly increased the expression levels of several genes related to taxol biosynthesis (Li et al., 2013). All these results indicated that the WRKY factor played an important role in taxol biosynthesis.

**Figure 10:**
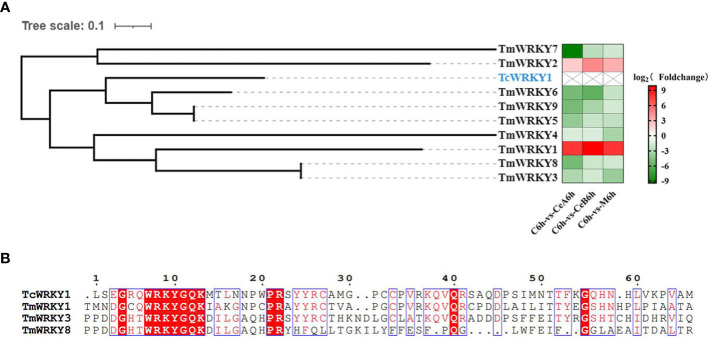
**(A)** hylogenetic analyses of WRKY coding genes. Protein marked in blue represent the WRKY that participated in the regulation of taxol biosynthesis pathway. The color density indicated log2 (Foldchange). Red indicated the upregulation of gene expression and green indicated the downregulation of gene expression. **(B)** Multiple sequence alignments of the WRKY domains of three TmWRKYs and TcWRKY1.

The transcriptome data showed that 83 unigenes were annotated to encode putative WRKY in Plant Transcription Factor Database. Sequence alignment and phylogenetic analysis of 9 differentially expressed WRKYs in the data (**Supplementary Material 4**) with TcWRKY1, which were known to be involved in taxol synthesis, revealed some homology of their nucleotide sequences (**Figure 10A**). Compared with control, 7 of the 9 genes encoding WRKY in three elicitation groups were all down-regulated, and other s were up-regulated. Additionally, the WRKY domain sequences alignments of three TmWRKYs and TcWRKY1 were shown in **Figure 10B**.

## Conclusion

The transcriptome of T. mairei cells unelicited and elicited by cerium and MJ were studied using Illumina sequencing technology. Analysis of the annotated unigenes revealed significant transcriptional complexity in *T. mairei* cells and provided more information for *T. mairei* cells response to cerium and MJ. After elicited by cerium and MJ , the downstream signal transduction stimulate the channels of MAPKs and other signal pathway components through superoxide anion. Then key TFs related to defense, such as WRKY, were triggered. TFs activated downstream defense pathways, such as JA signal transduction pathway and plant-pathogen interaction pathway, which finally increased taxol biosynthesis. Notably, Ce^3+^, Ce^4+^ and MJ can significantly increase taxol biosynthesis and release in *T. mairei.* However, taxol synthase coding genes had higher transcription levels by adding MJ, while Ce^3+^ and Ce^4+^ generally reduced the transcription levels of these genes. This might be that cerium could significantly induce the plant defensive response of taxus cells, leading to cell apoptosis. In conclusion, our transcriptome data will serve as an important public information platform for the comprehensive understanding of the cerium and MJ response networks and regulatory patterns, as well as the molecular mechanisms of cerium-mediated and MJ-mediated taxol biosynthesis in *T. mairei*.

## Data availability statement

The datasets presented in this study can be found in online repositories. The names of the repository/repositories and accession number(s) can be found below: https://www.ncbi.nlm.nih.gov/, SRR20217393.

## Author contributions

Conceptualization and design: NH, W-JG, S-TL, and P-PH. Methodology and formal analysis: NH, S-TL, Y-RL, and P-PH. Validation: S-TL, NH, JL and W-JG. Writing original draft preparation: NH, W-JG, JL, and P-PH. Plant cell culture: NH, JZ, Y-JW, H-HX and M-YL. Supervision and project administration: P-PH. Writing - review and editing: All authors. All authors contributed to the article and approved the submitted version.

## Funding

The authors are very grateful for the financial support from the National Key Research and Development Program of China (Grant No. 2021YFC2100800) and the Key Technologies R&D Program of Tianjin (No. 20YFZCSN00910).

## Conflict of interest

The authors declare that the research was conducted in the absence of any commercial or financial relationships that could be construed as a potential conflict of interest.

## Publisher’s note

All claims expressed in this article are solely those of the authors and do not necessarily represent those of their affiliated organizations, or those of the publisher, the editors and the reviewers. Any product that may be evaluated in this article, or claim that may be made by its manufacturer, is not guaranteed or endorsed by the publisher.

## Abbreviations

T. mairei, Taxus wallichiana var. mairei; RNA-Seq, RNA sequencing; TFs, Transcription factors; FDR, False Discovery Rate; DEG, Differential Expression Gene; Nr, NCBI Non-redundant Protein.

## References

[B1] Abdallah I. I. Pramastya H. van Merkerk R. (2019). Sukrasno and W.J. quax Metabolic engineering of bacillus subtilis toward taxadiene biosynthesis as the first committed step for taxol production. Front. Microbiol. 10, 218. doi: 10.3389/fmicb.2019.00218 30842758 PMC6391936

[B2] Andreasson E. Ellis B. (2010). Convergence and specificity in the arabidopsis MAPK nexus. Trends Plant Sci. 15, 106–113. doi: 10.1016/j.tplants.2009.12.001 20047850

[B3] Audic S. Claverie J. M. (1997). The significance of digital gene expression profiles. Genome Res. 7, 986–995. doi: 10.1101/gr.7.10.986 9331369

[B4] Bogolyubova I. Bogolyubov D. (2021). DAXX is a crucial factor for proper development of mammalian oocytes and early embryos. Int. J. Mol. Sci. 22 (3), 1313. doi: 10.3390/ijms22031313 33525665 PMC7866053

[B5] Chen S. A. Zhao B. Wang X. Yuan X. Wang Y. (2004). Promotion of the growth of crocus sativus cells and the production of crocin by rare earth elements. Biotechnol. Lett. 26, 27–30. doi: 10.1023/B:BILE.0000009455.31817.a5 15005147

[B6] Chini A. Fonseca S. Fernandez G. Adie B. Chico J. M. Lorenzo O.. (2007). The JAZ family of repressors is the missing link in jasmonate signalling. Nature 448, 666–671. doi: 10.1038/nature06006 17637675

[B7] Conesa A. Gotz S. Garcia-Gomez J. M. Terol J. Talon M. Robles M. (2005). Blast2GO: a universal tool for annotation, visualization and analysis in functional genomics research. Bioinformatics 21, 3674–3676. doi: 10.1093/bioinformatics/bti610 16081474

[B8] Cusido R. M. Onrubia M. Sabater-Jara A. B. Moyano E. Bonfill M. Goossens A.. (2014). A rational approach to improving the biotechnological production of taxanes in plant cell cultures of taxus spp. Biotechnol. Adv. 32, 1157–1167. doi: 10.1016/j.biotechadv.2014.03.002 24681092

[B9] Dai Y. L. Qin Q. L. Dai D. L. Kong L. S. Li W. Zha X. J.. (2009). Isolation and characterization of a novel cDNA encoding methyl jasmonate-responsive transcription factor TcAP2 from taxus cuspidata. Biotechnol. Lett. 31, 1801–1809. doi: 10.1007/s10529-009-0068-4 19565189

[B10] Dongming Ma G. P. Lei C. Ma L. Wang H. Guo Y. Chen J.. (2009). Isolation and characterization of AaWRKY1, an artemisia annua transcription factor that regulates the amorpha-4,11-diene synthase gene, a key gene of artemisinin biosynthesis. Plant C. Physiol. 50, 2146–2161. doi: 10.1093/pcp/pcp149 19880398

[B11] Fang W. S. Liang X. T. (2005). Recent progress in structure activity relationship and mechanistic studies of taxol analogues. Mini Rev. Med. Chem. 5, 1–12. doi: 10.2174/1389557053402837 15638787

[B12] Filipcik P. Latham S. L. Cadell A. L. Day C. L. Croucher D. R. Mace P. D. (2020). A cryptic tubulin-binding domain links MEKK1 to curved tubulin protomers. Proc. Natl. Acad. Sci. U.S.A. 117, 21308–21318. doi: 10.1073/pnas.2006429117 32817551 PMC7474687

[B13] Ge Z. Q. Yang S. Cheng J. S. Yuan Y. J. (2005). Signal role for activation of caspase-3-like protease and burst of superoxide anions during Ce4+-induced apoptosis of cultured taxus cuspidata cells. Biometals 18, 221–232. doi: 10.1007/s10534-005-0582-3 15984567

[B14] Gfeller A. Liechti R. Farmer E. E. (2010). Arabidopsis jasmonate signaling pathway. Sci. Signal 3, cm4. doi: 10.1126/scisignal.3109cm4 20159850

[B15] Grabherr M. G. Haas B. J. Yassour M. Levin J. Z. Thompson D. A. Amit I.. (2011). Full-length transcriptome assembly from RNA-seq data without a reference genome. Nat. Biotechnol. 29, 644–U130. doi: 10.1038/nbt.1883 21572440 PMC3571712

[B16] Hao D. C. Huang B. Yang L. (2008). Phylogenetic relationships of the genus taxus inferred from chloroplast intergenic spacer and nuclear coding DNA. Biol. Pharm. Bull. 31, 260–265. doi: 10.1248/bpb.31.260 18239284

[B17] Hong S. Y. Roze L. V. Linz J. E. (2013). Oxidative stress-related transcription factors in the regulation of secondary metabolism. Toxins (Basel) 5, 683–702. doi: 10.3390/toxins5040683 23598564 PMC3705287

[B18] Kakar K. U. Nawaz Z. Kakar K. Ali E. Almoneafy A. A. Ullah R.. (2017). Comprehensive genomic analysis of the CNGC gene family in brassica oleracea: novel insights into synteny, structures, and transcript profiles. BMC Genomics 18, 869. doi: 10.1186/s12864-017-4244-y 29132315 PMC5683364

[B19] Ketchum R. E. Gibson D. M. Croteau R. B. Shuler M. L. (1999). The kinetics of taxoid accumulation in cell suspension cultures of taxus following elicitation with methyl jasmonate. Biotechnol. Bioeng 62, 97–105. doi: 10.1002/(SICI)1097-0290(19990105)62:1<97::AID-BIT11>3.0.CO;2-C 10099517

[B20] Khosroushahi A. Y. Valizadeh M. Ghasempour A. Khosrowshahli M. Naghdibadi H. Dadpour M. R.. (2006). Improved taxol production by combination of inducing factors in suspension cell culture of taxus baccata. Cell Biol. Int. 30, 262–269. doi: 10.1016/j.cellbi.2005.11.004 16378737

[B21] Kuang X. J. Wang C. X. Zou L. Q. Li Y. Sun C. (2016). [Recent advances in biosynthetic pathway and synthetic biology of taxol]. Zhongguo Zhong Yao Za Zhi 41, 4144–4149. doi: 10.4268/cjcmm20162210 28933080

[B22] Lange C. Lyon H. DeMeo D. Raby B. Silverman E. K. Weiss S. T. (2003). A new powerful non-parametric two-stage approach for testing multiple phenotypes in family-based association studies. Hum. Hered 56, 10–17. doi: 10.1159/000073728 14614234

[B23] Lee E. K. Jin Y. W. Park J. H. Yoo Y. M. Hong S. M. Amir R.. (2010). Cultured cambial meristematic cells as a source of plant natural products. Nat. Biotechnol. 28, 1213–1217. doi: 10.1038/nbt.1693 20972422

[B24] Lenka S. K. Nims N. E. Vongpaseuth K. Boshar R. A. Roberts S. C. Walker E. L. (2015). Jasmonate-responsive expression of paclitaxel biosynthesis genes in taxus cuspidata cultured cells is negatively regulated by the bHLH transcription factors TcJAMYC1, TcJAMYC2, and TcJAMYC4. Front. Plant Sci. 6. doi: 10.3389/fpls.2015.00115 PMC4341510 25767476

[B25] Li X. C. Kang K. C. Huang X. Z. Fan Y. B. Song M. M. Huang Y. J.. (2020). [Genome-wide identification, phylogenetic analysis and expression profiling of the MKK gene family in arabidopsis pumila]. Yi Chuan 42, 403–421. doi: 10.16288/j.yczz.19-388 32312709

[B26] Li R. Q. Yu C. Li Y. R. Lam T. W. Yiu S. M. Kristiansen K.. (2009). SOAP2: an improved ultrafast tool for short read alignment. Bioinformatics 25, 1966–1967. doi: 10.1093/bioinformatics/btp336 19497933

[B27] Li Y. Y. Zhang H. X. Liang S. B. Chen X. L. Liu J. Y. Zhang Y.. (2022). Identification of CDPK gene family in solanum habrochaites and its function analysis under stress. Int. J. Mol. Sci. 23 (8), 4227. doi: 10.3390/ijms23084227 35457042 PMC9031491

[B28] Li S. Zhang P. Zhang M. Fu C. Yu L. (2013). Functional analysis of a WRKY transcription factor involved in transcriptional activation of the DBAT gene in taxus chinensis. Plant Biol. 15, 19–26. doi: 10.1111/j.1438-8677.2012.00611.x 22686366

[B29] Li S. T. Zhang P. Zhang M. Fu C. H. Zhao C. F. Dong Y. S.. (2012). Transcriptional profile of taxus chinensis cells in response to methyl jasmonate. BMC Genomics 13, 295. doi: 10.1186/1471-2164-13-295 22748077 PMC3414795

[B30] Mani S. D. Pandey S. Govindan M. Muthamilarasan M. Nagarathnam R. (2021). Transcriptome dynamics underlying elicitor-induced defense responses against septoria leaf spot disease of tomato (Solanum lycopersicum l.). Physiol. Mol. Biol. Plants 27, 873–888. doi: 10.1007/s12298-021-00970-y 33967469 PMC8055812

[B31] Mao R. Chen J. Chen Y. Guo Z. (2018). Identification of early jasmonate-responsive genes in taxus x media cells by analyzing time series digital gene expression data. Physiol. Mol. Biol. Plants 24, 715–727. doi: 10.1007/s12298-018-0527-2 30150849 PMC6103953

[B32] Ma Y. N. Xu D. B. Li L. Zhang F. Fu X. Q. Shen Q.. (2018). Jasmonate promotes artemisinin biosynthesis by activating the TCP14-ORA complex in artemisia annua. Sci. Adv. 4, eaas9357. doi: 10.1126/sciadv.aas9357 30627665 PMC6317983

[B33] Mithofer A. Schulze B. Boland W. (2004). Biotic and heavy metal stress response in plants: evidence for common signals. FEBS Lett. 566, 1–5. doi: 10.1016/j.febslet.2004.04.011 15147858

[B34] Mortazavi A. Williams B. A. Mccue K. Schaeffer L. Wold B. (2008). Mapping and quantifying mammalian transcriptomes by RNA-seq. Nat. Methods 5, 621–628. doi: 10.1038/nmeth.1226 18516045 PMC13303166

[B35] Mutanda I. Li J. Xu F. Wang Y. (2021). Recent advances in metabolic engineering, protein engineering, and transcriptome-guided insights toward synthetic production of taxol. Front. Bioeng Biotechnol. 9, 632269. doi: 10.3389/fbioe.2021.632269 33614616 PMC7892896

[B36] Nims E. Dubois C. P. Roberts S. C. Walker E. L. (2006). Expression profiling of genes involved in paclitaxel biosynthesis for targeted metabolic engineering. Metab. Eng. 8, 385–394. doi: 10.1016/j.ymben.2006.04.001 16793302

[B37] Patil R. A. Kolewe M. E. Normanly J. Walker E. L. Roberts S. C. (2012). Contribution of taxane biosynthetic pathway gene expression to observed variability in paclitaxel accumulation in taxus suspension cultures. Biotechnol. J. 7, 418–427. doi: 10.1002/biot.201100183 22095859 PMC3505991

[B38] Pertea G. Huang X. Q. Liang F. Antonescu V. Sultana R. Karamycheva S.. (2003). TIGR gene indices clustering tools (TGICL): a software system for fast clustering of large EST datasets. Bioinformatics 19, 651–652. doi: 10.1093/bioinformatics/btg034 12651724

[B39] Pop-Bica C. Ciocan C. A. Braicu C. Harangus A. Simon M. Nutu A.. (2022). Next-generation sequencing in lung cancer patients: A comparative approach in NSCLC and SCLC mutational landscapes. J. Pers. Med. 12 (3), 453. doi: 10.3390/jpm12030453 35330454 PMC8955273

[B40] Sharma T. R. Devanna B. N. Kiran K. Singh P. K. Arora K. Jain P.. (2018). Status and prospects of next generation sequencing technologies in crop plants. Curr. Issues Mol. Biol. 27, 1–36. doi: 10.21775/cimb.027.001 28885172

[B41] Sharma S. S. Kumar V. Dietz K. J. (2021). Emerging trends in metalloid-dependent signaling in plants. Trends Plant Sci. 26, 452–471. doi: 10.1016/j.tplants.2020.11.003 33257259

[B42] Shi M. Zhou W. Zhang J. Huang S. Wang H. Kai G. (2016). Methyl jasmonate induction of tanshinone biosynthesis in salvia miltiorrhiza hairy roots is mediated by JASMONATE ZIM-DOMAIN repressor proteins. Sci. Rep. 6, 20919. doi: 10.1038/srep20919 26875847 PMC4753458

[B43] Sun Y. Liu D. L. Yu Z. Q. Zhang Q. Bai J. Sun D. Y. (2003). An apoplastic mechanism for short-term effects of rare earth elements at lower concentrations. Plant Cell Environ. 26, 887–896. doi: 10.1046/j.1365-3040.2003.01021.x 12803616

[B44] Tatusov R. L. Fedorova N. D. Jackson J. D. Jacobs A. R. Kiryutin B. Koonin E. V.. (2003). The COG database: an updated version includes eukaryotes. BMC Bioinf. 4, 41. doi: 10.1186/1471-2105-4-41 PMC222959 12969510

[B45] Thines B. Katsir L. Melotto M. Niu Y. Mandaokar A. Liu G.. (2007). JAZ repressor proteins are targets of the SCF(COI1) complex during jasmonate signalling. Nature 448, 661–665. doi: 10.1038/nature05960 17637677

[B46] Wang T. Li L. Zhuang W. Zhang F. Shu X. Wang N.. (2021). Recent research progress in taxol biosynthetic pathway and acylation reactions mediated by taxus acyltransferases. Molecules 26 (10), 2855. doi: 10.3390/molecules26102855 34065782 PMC8151764

[B47] Wang W. Yang Y. Zheng X. D. Huang S. Q. Guo L. Kong J. Q.. (2013). [The advance in synthetic biology: towards a microbe-derived paclitaxel intermediates]. Yao Xue Xue Bao 48, 187–192. 23672014

[B48] Wang T. Zhang F. J. Zhuang W. B. Shu X. C. Wang Z. (2019). Metabolic variations of flavonoids in leaves of t. media and t. mairei obtained by UPLC-ESI-MS/MS. Molecules 24 (18), 3323. doi: 10.3390/molecules24183323 31547329 PMC6767174

[B49] Wen G. Qu X. X. Wang D. Chen X. X. Tian X. C. Gao F.. (2016). Recent advances in design, synthesis and bioactivity of paclitaxel-mimics. Fitoterapia 110, 26–37. doi: 10.1016/j.fitote.2016.02.010 26906104

[B50] Wu K. L. Guo Z. J. Wang H. H. Li J. (2005). The WRKY family of transcription factors in rice and arabidopsis and their origins. DNA Res. 12, 9–26. doi: 10.1093/dnares/12.1.9 16106749

[B51] Wu H. G. Yuan Y.J.J.C.R.E. (2000). Effect of Ce(NO_3)_3 on the synthesis of taxol in taxus Chinese cell culture. Chinese Rare Earths 21 (2).

[B52] Xiao X. H. Yang M. Sui J. L. Qi J. Y. Fang Y. J. Hu S. N.. (2017). The calcium-dependent protein kinase (CDPK) and CDPK-related kinase gene families in hevea brasiliensis-comparison with five other plant species in structure, evolution, and expression. FEBS Open Bio 7, 4–24. doi: 10.1002/2211-5463.12163 PMC5221434 28097084

[B53] Xu Y. H. Wang J. W. Wang S. Wang J. Y. Chen X.Y.J.P.P. (2004). Characterization of GaWRKY1, a cotton transcription factor that regulates the sesquiterpene synthase gene (+)-δ-Cadinene synthase-a. Plant Physiology 135, 507–515. doi: 10.1104/pp.104.038612 15133151 PMC429402

[B54] Yamaguchi K. Shirakabe K. Shibuya H. Irie K. Oishi I. Ueno N.. (1995). Identification of a member of the MAPKKK family as a potential mediator of TGF-beta signal transduction. Science 270, 2008–2011. doi: 10.1126/science.270.5244.2008 8533096

[B55] Yang S. Ge Z. Yuan Y. Engineering (2006). ERK-like MAP kinase regulated by O_2~-·during ce ~(4+)-induced apoptois of cultured taxus cuspidata cells. J. Chem. Eng. 57, 902–907.

[B56] Yingjin Y. Guowu H. Chuangu W. Ying J. Panwen S. (1998). Effect of la, ce on taxus cuspidata cell growth, biosynthesis and release of taxol. J. Chinese Rare Earth Society 16 (1).

[B57] Yuan Y.J.J.O.I.B. (2006). Ce4+ induced down-regulation of ERK-like MAPK and activation of nucleases during the apoptosis of cultured taxus cuspidata cells. J. Inorg. Biochem. 100 (2), 167–177. doi: 10.1016/j.jinorgbio.2005.10.001 16293312

[B58] Yuan Y. J. Li J. C. Ge Z. Q. Wu J.C.J.J.O.M.C.B.E. (2002). Superoxide anion burst and taxol production induced by Ce4+ in suspension cultures of taxus cuspidata. J. Mol. Catal., B Enzym 18, 251–260. doi: 10.1016/S1381-1177(02)00103-0

[B59] Yuan Y. J. Li C. Hu Z. D. Wu J. C. (2001). Signal transduction pathway for oxidative burst and taxol production in suspension cultures of taxus chinensis var. mairei induced by oligosaccharide from fusarium oxysprum. Enzyme Microb. Tech 29, 372–379. doi: 10.1016/S0141-0229(01)00406-9

[B60] Yu C. Guo H. Zhang Y. Song Y. Pi E. Yu C.. (2017). Identification of potential genes that contributed to the variation in the taxoid contents between two taxus species (Taxus media and taxus mairei). Tree Physiol. 37, 1659–1671. doi: 10.1093/treephys/tpx091 28985439

[B61] Zhang M. Chen Y. Nie L. Jin X. Liao W. Zhao S.. (2018). Transcriptome-wide identification and screening of WRKY factors involved in the regulation of taxol biosynthesis in taxus chinensis. Sci. Rep. 8, 5197. doi: 10.1038/s41598-018-23558-1 29581461 PMC5980082

[B62] Zhang D. Facchinetti V. Wang X. Huang Q. Qin J. Su B. (2006). Identification of MEKK2/3 serine phosphorylation site targeted by the toll-like receptor and stress pathways. EMBO J. 25, 97–107. doi: 10.1038/sj.emboj.7600913 16362041 PMC1356356

[B63] Zhang M. Li S. T. Nie L. Chen Q. P. Xu X. P. Yu L. J.. (2015). Two jasmonate-responsive factors, TcERF12 and TcERF15, respectively act as repressor and activator of tasy gene of taxol biosynthesis in taxus chinensis. Plant Mol. Biol. 89, 463–473. doi: 10.1007/s11103-015-0382-2 26445975

[B64] Zhang M. Zhang S. (2022). Mitogen-activated protein kinase cascades in plant signaling. J. Integr. Plant Biol. 64, 301–341. doi: 10.1111/jipb.13215 34984829

[B65] Zhao J. Peng S. Cui H. Li P. Li T. Liu L.. (2022). Dynamic expression, differential regulation and functional diversity of the CNGC family genes in cotton. Int. J. Mol. Sci. 23 (4), 2041. doi: 10.3390/ijms23042041 35216157 PMC8878070

