# Supplementary material for: Transcription level differences in Taxus wallichiana var. mairei elicited by Ce3+, Ce4+ and methyl jasmonate
Source: Front Plant Sci. 2022 Nov 10;13:1040596. doi: 10.3389/fpls.2022.1040596 (PMC9685566; doi:10.3389/fpls.2022.1040596)
Supplement: Supplementary file 1 [file DataSheet_1.docx]

Transcription level differences in Taxus wallichiana var. mairei elicited by Ce3+, Ce4+ and methyl jasmonate

Supplementary material 1


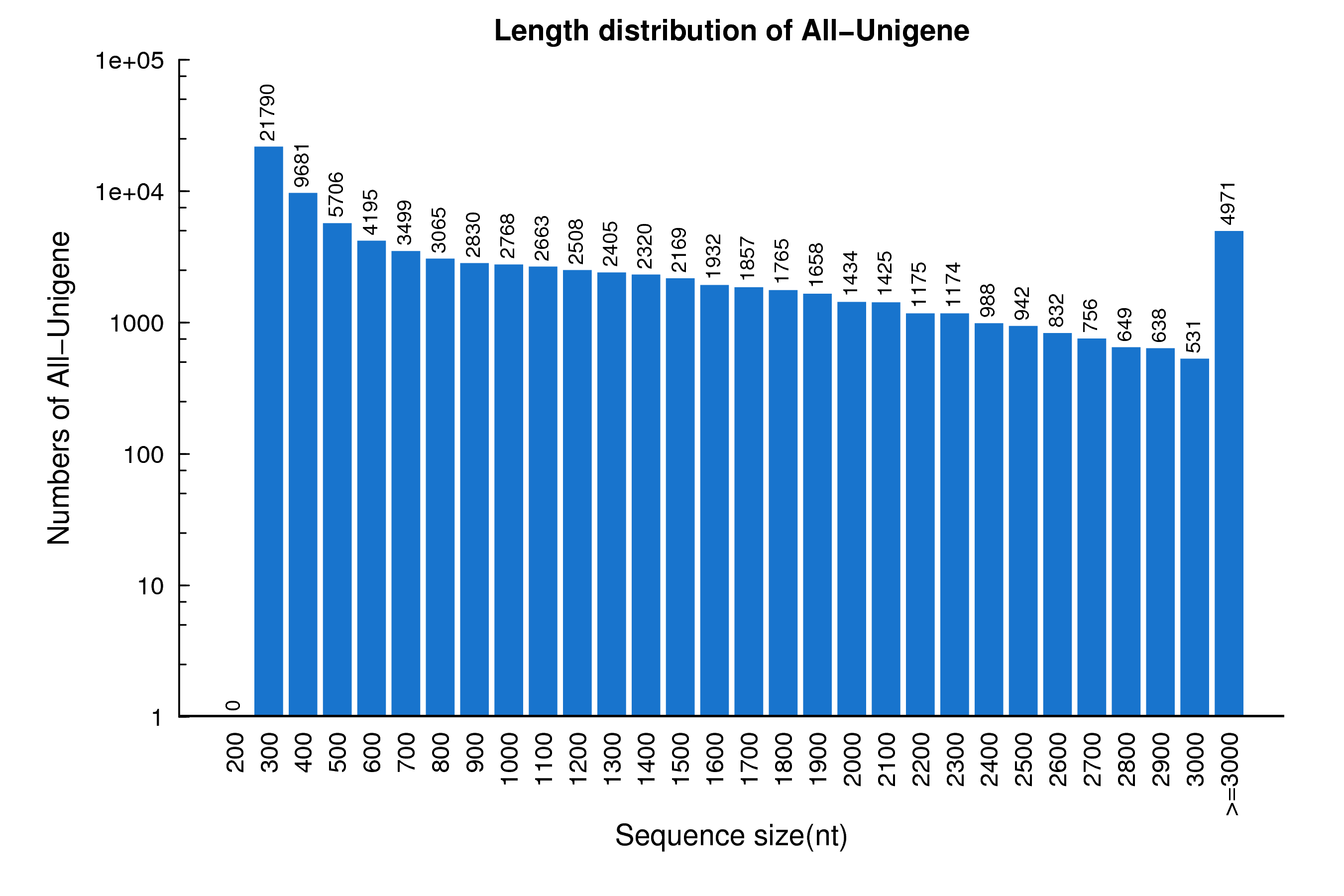


Fig.S1 Length distribution of all assembled unigenes.

Supplementary material 2

TableS1 The annotation of unigenes basing on various databases.

| **Database** | **Annotated Unigenes** |
| --- | --- |
| NR | 51,400 |
| Swiss-Prot | 41,387 |
| KEGG | 35,262 |
| COG | 21,639 |
| GO | 35,031 |
| ALL of Annotated Unigenes | 51,516 |

Supplementary material 3


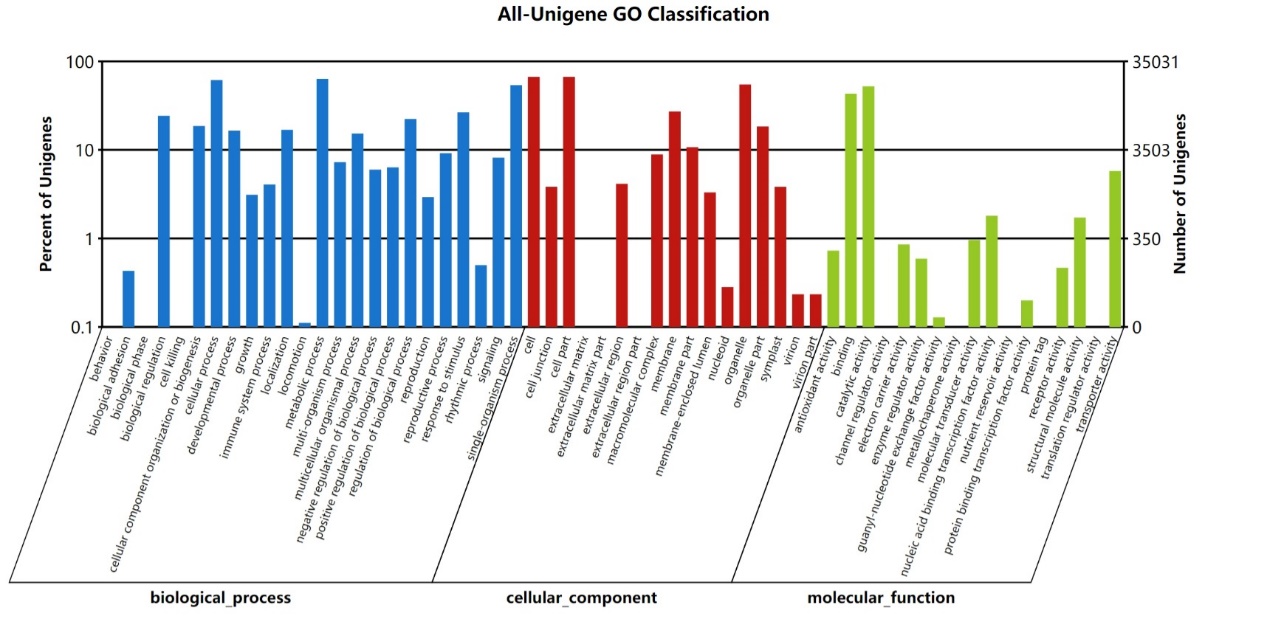


Fig.S2 GO classification of all unigenes.

Supplementary material 4

| Name | geneID | Best hit in A.thaliana | Description for the best hit |
| --- | --- | --- | --- |
| TmWRKY1 | CL1053.Contig8_All | AT5G15130.1 | WRKY DNA-binding protein 72 |
| TmWRKY2 | Unigene32980_All | AT5G13080.1 | WRKY DNA-binding protein 75 |
| TmWRKY3 | CL4152.Contig3_All | AT4G11070.2 | WRKY family protein |
| TmWRKY4 | Unigene18409_All | AT2G30590.1 | WRKY DNA-binding protein 21 |
| TmWRKY5 | CL2609.Contig4_All | AT4G01720.1 | WRKY family protein |
| TmWRKY6 | CL2609.Contig3_All | AT4G31800.1 | WRKY DNA-binding protein 18 |
| TmWRKY7 | CL3606.Contig1_All | AT4G26640.2 | WRKY family protein |
| TmWRKY8 | CL4152.Contig4_All | AT4G11070.2 | WRKY family protein |
| TmWRKY9 | CL2609.Contig2_All | AT4G01720.1 | WRKY family protein |
